# Disperse fine equiaxed alpha alumina nanoparticles with narrow size distribution synthesised by selective corrosion and coagulation separation

**DOI:** 10.1038/srep11575

**Published:** 2015-07-13

**Authors:** Sanxu Pu, Lu Li, Ji Ma, Fuliang Lu, Jiangong Li

**Affiliations:** 1 Institute of Materials Science & Engineering, Lanzhou University, Lanzhou 730000, China

## Abstract

Disperse fine equiaxed *α*-Al_2_O_3_ nanoparticles with narrow size distribution are important materials in nanotechnology and nanomaterials, but syntheses of disperse fine equiaxed *α*-Al_2_O_3_ nanoparticles usually result in fine *γ*-Al_2_O_3_ nanoparticles or large *α*-Al_2_O_3_ nanoparticles larger than 15 nm. *α*-Al_2_O_3_ has a higher surface energy than *γ*-Al_2_O_3_ and becomes thermodynamically not stable with respect to *γ*-Al_2_O_3_ at specific surface areas larger than 100 m^2^/g (at sizes smaller than 15 nm for spherical particles) at room temperature. So disperse fine equiaxed *α*-Al_2_O_3_ nanoparticles smaller than 15 nm with narrow size distribution are extremely difficult to synthesise. Here we show the successful synthesis of disperse fine equiaxed *α*-Al_2_O_3_ nanoparticles with average sizes below 10 nm and narrow size distribution by selective corrosion and refined fractionated coagulation separation. An almost fully dense nanocrystalline *α*-Al_2_O_3_ ceramic with a relative density of 99.5% and an average grain size of 60 nm can be sintered from disperse fine equiaxed *α*-Al_2_O_3_ nanoparticles with narrow size distribution.

Disperse fine equiaxed *α*-Al_2_O_3_ nanoparticles (NPs) are important materials for applications such as cancer therapy^1^, abrasive polishing^2^ and nanocomposite materials^3^. Disperse fine equiaxed *α*-Al_2_O_3_ NPs with narrow size distribution are essential raw materials for sintering nanocrystalline *α*-Al_2_O_3_ ceramic which may exhibit low temperature ductility like nanocrystalline CaF_2_ and TiO_2_ ceramics with an average grain size of 8 nm showing large plastic deformations at 80 and 180 °C respectively^4^ and nanocrystalline TiO_2_ ceramic with an average grain size of 40 nm showing a large true strain of −0.6 at 800 °C^5^. Many efforts have been made to synthesise disperse fine equiaxed *α*-Al_2_O_3_ NPs with sizes below 15 nm and narrow size distribution^6^,^7^,^8^,^9^,^10^,^11^,^12^,^13^,^14^,^15^. Johnston *et al*. reported *γ*-Al_2_O_3_ NPs of 5–14 nm in size prepared by reactive laser ablation of aluminum in oxygen, but upon transformation into *α*-Al_2_O_3_ at 1200 °C, the particles grew to large particles of about 52 nm^6^. Noguchi *et al*. synthesised fine *γ*-Al_2_O_3_ NPs of about 6 nm in supercritical water by continuous hydrothermal flow reaction system^7^. Li *et al*. prepared vermicular *α*-Al_2_O_3_ particles with sizes of about 100 nm agglomerated from primary particles of about 10 nm by precipitation method using *α*-Al_2_O_3_ seeding and calcination at 900 °C^8^. Das *et al*. synthesised porous *α*-Al_2_O_3_ powders of the hard-agglomerated particles with primary particle sizes of about 20 nm by the thermal decomposition of an aqueous solution of aluminum nitrate and sucrose and calcination at 600 °C^9^. Laine *et al*. converted *θ*-Al_2_O_3_ NPs to 29 nm (86% *α* and 14% *θ*) Al_2_O_3_ NPs by liquid-feed flame spray pyrolysis and sintered a 99.5% dense *α*-Al_2_O_3_ ceramic with a grain size of about 350 nm from the Al_2_O_3_ NPs^10^. Zhang *et al*. prepared *α*-Al_2_O_3_ nanostructures of about 150 nm in size consisting of nanorods of about 15 nm in diametre and about 150 nm in length by calcining fine boehmite powders at 1000 °C^11^. Yoo *et al*. reported vermicular *α*-Al_2_O_3_ particles with primary particle sizes of about 35 nm synthesised by vapor-phase hydrolysis of AlCl_3_ and calcination at 1200 °C^12^. Karagedov *et al*. prepared disperse equiaxed *α*-Al_2_O_3_ NPs with an average size of 25 nm by ball-milling *α*-Al_2_O_3_ powders and boiling the ball-milled *α*-Al_2_O_3_ powders in HCl^13^ and *α*-Al_2_O_3_ NPs of about 50 nm by precipitation method using 25 nm *α*-Al_2_O_3_ NP seeding and calcination at 930 °C^14^. Borsella *et al*. synthesised vermicular *α*-Al_2_O_3_ NPs with primary particle sizes of about 15 nm by laser synthesis from gaseous precursors^15^. So syntheses of disperse fine equiaxed *α*-Al_2_O_3_ NPs usually result in fine *γ*-Al_2_O_3_ NPs^6^,^7^ or large *α*-Al_2_O_3_ NPs larger than 15 nm^8^,^9^,^10^,^11^,^12^,^13^,^14^,^15^.

*α*-Al_2_O_3_ (corundum) is the thermodynamically stable phase of bulk Al_2_O_3_ at common pressure and temperature conditions. Simulation and experimental studies showed that *α*-Al_2_O_3_ has a higher surface energy (2.64 J/m^2^) than *γ*-Al_2_O_3_ (1.67 J/m^2^) and becomes thermodynamically not stable with respect to *γ*-Al_2_O_3_ at specific surface areas larger than 125 m^2^/g (at specific surface areas larger than 100 m^2^/g at room temperature)^16^,^17^ (specific surface areas of 125 and 100 m^2^/g correspond to diametres of 12 and 15 nm for spherical particles respectively). Perrotta *et al*. synthesised *α*-Al_2_O_3_ particles with a specific surface area of 150 m^2^/g by the dehydration of diaspore to *α*-Al_2_O_3_^18^,^19^ and attributed the formation of the high surface area *α*-Al_2_O_3_ NPs to the chemisorbed H_2_O which could stabilise the high surface area of *α*-Al_2_O_3_ NPs^18^, but these high surface area *α*-Al_2_O_3_ particles have a platy morphology^20^. So the synthesis of disperse fine equiaxed *α*-Al_2_O_3_ NPs smaller than 15 nm with narrow size distribution is extremely difficult and remains a challenge so far.

In this article, we present the successful synthesis of disperse fine equiaxed *α*-Al_2_O_3_ NPs with different average particle sizes below 10 nm and narrow size distribution widths by the refined fractionated coagulation separations of disperse equiaxed *α*-Al_2_O_3_ NPs with a wide size distribution width. Disperse equiaxed *α*-Al_2_O_3_ NPs with a wide size distribution width were prepared by removing the matrixes in the *α*-Al_2_O_3_-NPs-embedded composites through selective corrosion. The *α*-Al_2_O_3_-NPs-embedded composite powders were synthesised by mechanochemical method. Our primary sintering experiments demonstrate that green compacts of the *α*-Al_2_O_3_ NPs with an average particle size of 7.9 nm and a size distribution width of 4–14 nm were sintered to an almost fully dense nanocrystalline *α*-Al_2_O_3_ ceramic with a relative density of 99.5% and an average grain size of 60 nm by a non-optimised two-step pressureless sintering.

## Results

**Figure 1:**
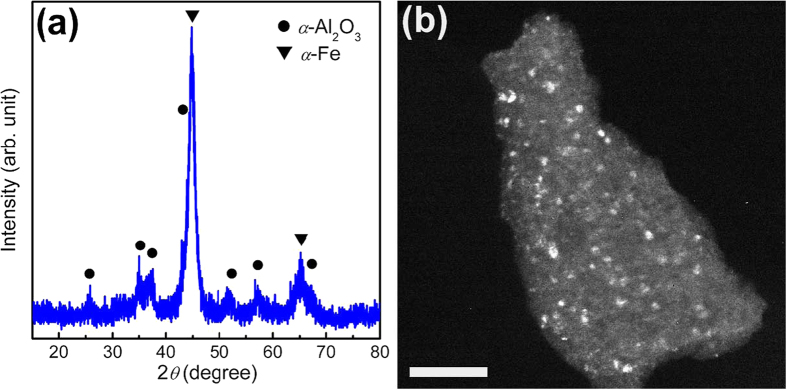
Composites of *α*-Al_2_O_3_ NPs embedded in Fe matrix. (**a**) XRD pattern of the composite powders obtained by ball-milling stoichiometric mixtures of Al and Fe_2_O_3_ powders (according to 2Al + Fe_2_O_3_ = 2Fe + Al_2_O_3_) at a BPR of 20:1 and a main disk rotation speed of 300 rpm for 20 h, showing coexistence of *α*-Al_2_O_3_ and *α*-Fe. (**b**) Dark-field TEM image of the ball-milled composite powders showing fine equiaxed *α*-Al_2_O_3_ NPs (bright) in the composite. Scale bar, 100 nm.

To prepare disperse fine equiaxed *α*-Al_2_O_3_ NPs, *α*-Al_2_O_3_-NPs-embedded composite powders were synthesised at first by mechanochemical method. Stoichiometric mixtures of Al and Fe_2_O_3_ powders (according to Fe_2_O_3_ + 2Al = 2Fe + Al_2_O_3_) were milled under the optimised ball milling conditions [a main disk rotation speed of 300 revolutions per minute (rpm), a ball-to-powder ratio (BPR) of 20:1 and a milling duration of 20 h] [see Methods and Supplementary Information] and characterized by x-ray diffraction (XRD) and transmission electron microscopy (TEM). The XRD pattern of the ball-milled powders shows the overlapped XRD patterns of *α*-Al_2_O_3_ and *α*-Fe without any other diffraction peaks (Fig. 1a), indicating that the ball-milled powders consist of *α*-Al_2_O_3_ and *α*-Fe phases. The dark-field TEM observations reveal equiaxed *α*-Al_2_O_3_ NPs of 2–250 nm in size in the ball-milled powders. The dark-field TEM image of the ball-milled powders in Fig. 1b shows that *α*-Al_2_O_3_ NPs (bright) in the ball-milled powders are 3 to 20 nm in size and equiaxed in shape. The XRD and dark-field TEM analyses demonstrate that the ball-milled powders are the composites of *α*-Al_2_O_3_ NPs embedded in the Fe matrix. The volume fraction of *α*-Al_2_O_3_ in the composites, estimated from the stoichiometry of 2Al + Fe_2_O_3_ = 2Fe + Al_2_O_3_, is about 64%. *α*-Al_2_O_3_-NPs-embedded composite powders can also be prepared using Al and CoO powders as starting powders by mechanochemical method.

**Figure 2:**
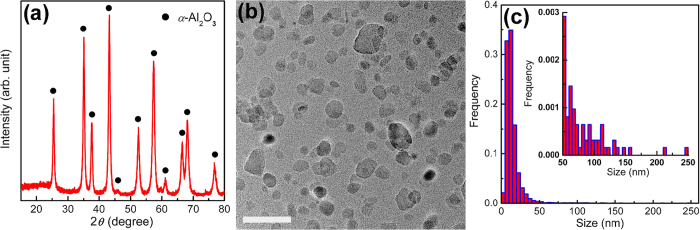
*α*-Al_2_O_3_ NPs obtained by removing the Fe matrix in the composites. (**a**) XRD pattern of the *α*-Al_2_O_3_ NPs obtained by removing the Fe matrix in the composites of *α*-Al_2_O_3_ NPs embedded in the Fe matrix through selective corrosion, showing a typical XRD pattern of *α*-Al_2_O_3_. (**b**) TEM image of the *α*-Al_2_O_3_ NPs obtained by removing the Fe matrix in the composites, revealing disperse equiaxed NPs. Scale bar, 50 nm. (**c**) Size distribution histogram (inset: enlarged part for large particles) of the *α*-Al_2_O_3_ NPs obtained by removing the Fe matrix in the composites, showing that most of *α*-Al_2_O_3_ NPs are smaller than 15 nm.

To obtain disperse fine equiaxed *α*-Al_2_O_3_ NPs, the matrixes in the *α*-Al_2_O_3_-NPs-embedded composites were removed through selective corrosion. The composite powders of *α*-Al_2_O_3_ NPs embedded in the Fe matrix prepared by high-energy ball milling were corroded with hydrochloric acid at room temperature and at 120 °C (Methods and Supplementary Information). The powders obtained by selective corrosion were analysed by XRD, TEM, energy dispersive x-ray spectroscopy (EDS) and inductively coupled plasma-atomic emission spectrometry (ICP-AES). The powders yield a typical XRD pattern of *α*-Al_2_O_3_ without any additional diffraction peaks (Fig. 2a), indicating that the powders are pure *α*-Al_2_O_3_. This phase identification was supported by the selected area electron diffraction (SAED) pattern of the powders obtained by selective corrosion (Supplementary Fig. S1a). The TEM image in Fig. 2b and the low magnification TEM image in Supplementary Fig. S1b reveal that the powders obtained by selective corrosion are disperse equiaxed *α*-Al_2_O_3_ NPs of sizes ranging from 3 to 200 nm without agglomeration. The average particle size and size distribution of the *α*-Al_2_O_3_ NPs were statistically determined from the TEM observations; and the size distribution histogram of the *α*-Al_2_O_3_ NPs is shown in Fig. 2c. According to the size distribution histogram of the *α*-Al_2_O_3_ NPs (Fig. 2c), most of *α*-Al_2_O_3_ NPs (~69%) are smaller than 15 nm whereas few of *α*-Al_2_O_3_ NPs (~31%) are larger than 15 nm, and the average particle size and size distribution width of the *α*-Al_2_O_3_ NPs are 14.3 and 2–250 nm respectively. The purity of the disperse equiaxed *α*-Al_2_O_3_ NPs, determined by EDS and ICP-AES analyses (Supplementary Fig. S2 and Supplementary Tables S1 and S2), is 99.6% (mass percent) though the starting Al and Fe_2_O_3_ powders only have a purity of 99.0% (mass percent).

**Figure 3:**
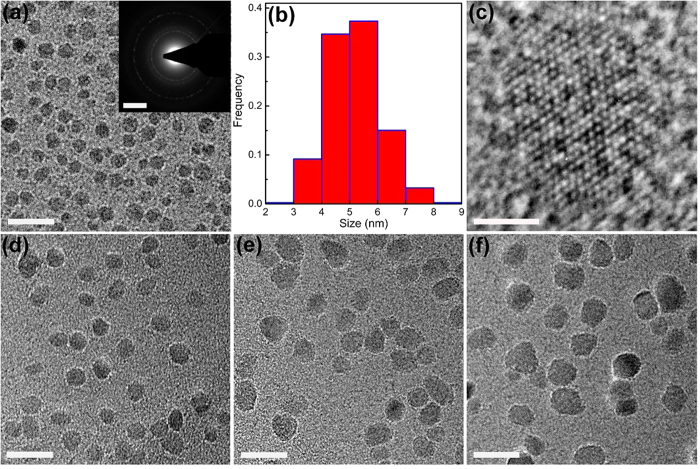
*α*-Al_2_O_3_ NPs with different average particle sizes and narrow size distribution widths. (**a**,**b**) TEM image (scale bar, 20 nm; inset: SAED pattern, scale bar, 5 nm^−1^) (**a**) and size distribution histogram (**b**) of the *α*-Al_2_O_3_ NPs obtained by refined fractionated coagulation separation with 1.4 mol/L HCl, showing disperse fine equiaxed *α*-Al_2_O_3_ NPs with an average particle size of 5.2 nm and a size distribution width of 2–9 nm. (**c**) HRTEM image (the beam direction in [0001]; scale bar, 2 nm) of a single *α*-Al_2_O_3_ NP among the *α*-Al_2_O_3_ NPs with an average size of 5.2 nm, showing the hexagonal lattice of *α*-Al_2_O_3_. (**d**–**f**) TEM images (scale bar, 20 nm) of the *α*-Al_2_O_3_ NPs with average particle sizes of 6.5 (d), 7.9 (**e**) and 9.6 nm (**f**) obtained by refined fractionated coagulation separation with 1.2, 1.0 and 0.8 mol/L HCl respectively. Low magnification TEM images of the *α*-Al_2_O_3_ NPs with average sizes of 5.2, 6.5, 7.9 and 9.6 nm are shown in Supplementary Fig. S3a-d.

Disperse equiaxed *α*-Al_2_O_3_ NPs, obtained by removing the Fe matrix in the *α*-Al_2_O_3_-NPs-embedded composites through selective corrosion, have a small average particle size of 14.3 nm but a quite wide size distribution width from 2 to 250 nm (Fig. 2c). For many applications, for example, sintering dense nanocrystalline ceramics, a narrow particle size distribution width is desired because large particles will grow at the expense of small particles (Ostwald ripening) during sintering^21^,^22^,^23^, resulting in coarse-grained ceramics rather than expected nanocrystalline ceramics. To obtain disperse fine equiaxed *α*-Al_2_O_3_ NPs with a narrow size distribution width, the disperse equiaxed *α*-Al_2_O_3_ NPs with a wide size distribution width of 2–250 nm should be size-selectively separated. Fractionated coagulation was applied to separate Au NPs of 7.5 and 80 nm using NaNO_3_ as coagulating agent^24^. The critical coagulation concentration decreases when the particle size increases^24^. The disperse equiaxed *α*-Al_2_O_3_ NPs with a wide size distribution width of 2–250 nm were size-selectively separated by refined fractionated coagulation (Methods). Here refined fractionated coagulation refers to fractionated coagulation by decreasing the concentration of coagulating agent in a small interval to decrease size distribution widths of coagulation-separated NPs. Hydrochloric acid was used as coagulating agent for the size-selective separations of *α*-Al_2_O_3_ NPs. The particle size of coagulation increases when the HCl concentration decreases for disperse equiaxed *α*-Al_2_O_3_ NPs. By stepwise decreasing HCl concentration from 1.4 to 0.8 mol/L at a step of 0.2 mol/L, namely at HCl concentrations of 1.4, 1.2, 1.0 and 0.8 mol/L, disperse fine equiaxed *α*-Al_2_O_3_ NPs with an average size of 5.2 nm and a size distribution width of 2–9 nm, an average size of 6.5 nm and a size distribution width of 3–11 nm, an average size of 7.9 nm and a size distribution width of 4–14 nm as well as an average size of 9.6 nm and a size distribution width of 5–15 nm were separated by refined fractionated coagulation respectively (Table 1). The TEM images of the *α*-Al_2_O_3_ NPs with an average size of 5.2 nm (Fig. 3a and Supplementary Fig. S3a) show that the *α*-Al_2_O_3_ NPs are fully disperse without any agglomeration, 3 to 8 nm in size and equiaxed in shape. The SAED pattern in the inset of Fig. 3a supports the phase identification of *α*-Al_2_O_3_ by XRD analysis. The size distribution width of the *α*-Al_2_O_3_ NPs with an average size of 5.2 nm (Fig. 3b) is narrow (2–9 nm), compared with that before the size-selective separation (2–250 nm) in Fig. 2c. The high resolution TEM (HRTEM) image in Fig. 3c shows the two-dimensional hexagonal lattice image of a single *α*-Al_2_O_3_ NP among the *α*-Al_2_O_3_ NPs with an average size of 5.2 nm along the [0001] direction. The TEM images of the *α*-Al_2_O_3_ NPs with average particle sizes (and size distribution widths) of 6.5 (3–11), 7.9 (4–14) and 9.6 (5–15) nm in Fig. 3d–f and Supplementary Fig. S3b-d reveal disperse fine equiaxed *α*-Al_2_O_3_ NPs with narrow size distribution widths (Supplementary Fig. S4). The XRD analysis of the *α*-Al_2_O_3_ NPs with an average size of 7.9 nm shows the broad diffraction peaks of pure *α*-Al_2_O_3_ (Supplementary Fig. S5). The average grain size estimated from the XRD peak widths using Scherrer equation is 7.5 nm, close to the average particle size of the *α*-Al_2_O_3_ NPs (7.9 nm) determined statistically by TEM analysis. The specific surface area of the *α*-Al_2_O_3_ NPs with an average size of 7.9 nm, measured by N_2_ adsorption at 77 K using the Brunauer-Emmett-Teller (BET) method (Supplementary Fig. S6), is 170 m^2^/g.

**Figure 4:**
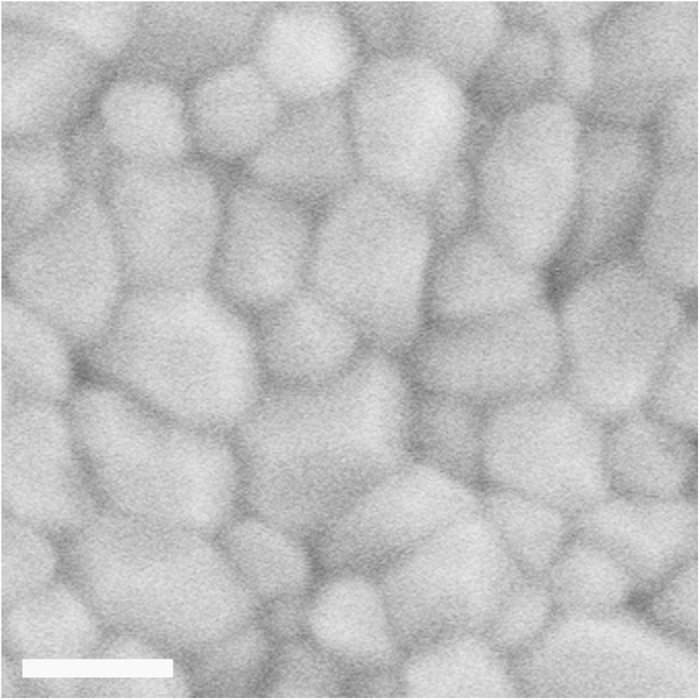
Microstructure of almost fully dense nanocrystalline *α*-Al_2_O_3_ ceramic with an average grain size of 60 nm. SEM cross-sectional image of the sintered body of a green compact pressed from the disperse fine equiaxed *α*-Al_2_O_3_ NPs with an average particle size of 7.9 nm and a size distribution width of 4–14 nm at 600 MPa and sintered by a non-optimised two-step pressureless sintering (heating to 1,230 °C without hold and decreasing to 1,080 °C with a 40 h hold in air) (after additional thermal etching). Scale bar, 100 nm. The sintered nanocrystalline *α*-Al_2_O_3_ ceramic has a relative density of 99.5%, an average grain size of 60 nm and a grain size distribution width of 20–130 nm. A low magnification SEM image of the sintered nanocrystalline *α*-Al_2_O_3_ ceramic is shown in Supplementary Fig. S7.

To check the sintering activity of disperse fine equiaxed *α*-Al_2_O_3_ NPs with narrow size distribution widths (separated by refined fractionated coagulation), our disperse fine equiaxed *α*-Al_2_O_3_ NPs were pressed into green compacts and sintered (Methods). Chen *at al*. developed the two-step pressureless sintering method to suppress the final-stage grain growth and sintered dense nanocrystalline Y_2_O_3_ ceramic with a grain size of 60 nm^25^. Our *α*-Al_2_O_3_ green compacts pressed from the *α*-Al_2_O_3_ NPs with an average particle size of 7.9 nm and a size distribution width of 4–14 nm at 600 MPa were sintered in air by a two-step sintering (heating to 1,230 °C without hold, then decreasing to 1,080 °C with a 40 h hold). The SEM observations and Archimedes measurements reveal that the sintered bodies have a relative density of 99.5%, an average grain size of 60 nm and a grain size distribution width of 20–130 nm (Fig. 4 and Supplementary Fig. S7). This two-step pressureless sintering process (1,230 °C–1,080 °C for 40 h) was not optimised, yet the sintered nanocrystalline *α*-Al_2_O_3_ ceramic exhibits a relative density as high as 99.5% and an average grain size as small as 60 nm.

## Discussion

The disperse fine equiaxed *α*-Al_2_O_3_ NPs separated by refined fractionated coagulation have narrow size distribution widths and average particle sizes below 10 nm, much smaller than the particle sizes of disperse equiaxed *α*-Al_2_O_3_ NPs achieved so far (25 nm *α*-Al_2_O_3_ NPs prepared by ball-milling and HCl boiling^14^ or 29 nm (86% *α* and 14% *θ*) Al_2_O_3_ NPs synthesised by liquid-feed flame spray pyrolysis^10^). The specific surface area of the *α*-Al_2_O_3_ NPs with an average size of 7.9 nm (170 m^2^/g) is close to a specific surface area of 178 m^2^/g, estimated from the size distribution histogram of the *α*-Al_2_O_3_ NPs with an average size of 7.9 nm (Supplementary Fig. S4) by assuming a spherical shape for the *α*-Al_2_O_3_ NPs and much higher than those of high surface area platy *α*-Al_2_O_3_ particles synthesised by the dehydration of diaspore (150 m^2^/g)^18^,^19^, (86% *α*) Al_2_O_3_ NPs synthesised by liquid-feed flame spray pyrolysis (40–60 m^2^/g)^10^ and *α*-Al_2_O_3_ NPs prepared by ball-milling and HCl boiling (57 m^2^/g)^14^. It is also much higher than 100 m^2^/g above which *α*-Al_2_O_3_ becomes thermodynamically not stable with respect to *γ*-Al_2_O_3_ at room temperature^17^. Moreover, the refined fractionated coagulation separation method is more efficient and much simpler than other size-selective separation methods such as gel electrophoresis^26^ and density gradients^27^ and can be scaled up for large-scale separations of *α*-Al_2_O_3_ NPs with narrow size distribution widths and average particle sizes below 10 nm (and for large-scale separations of *α*-Al_2_O_3_ NPs with narrow size distribution widths and average particle sizes above 10 nm as well, see Supplementary Fig. S3e-h) from *α*-Al_2_O_3_ NPs with a wide size distribution width.

*α*-Al_2_O_3_ in bulk form is thermodynamically stable at common pressure and temperature conditions. However, *α*-Al_2_O_3_ has a higher surface energy than *γ*-Al_2_O_3_, equiaxed *α*-Al_2_O_3_ NPs smaller than 15 nm are thermodynamically not stable (equiaxed *γ*-Al_2_O_3_ NPs smaller than 15 nm are thermodynamically stable) at room temperature^17^. The formation of fine equiaxed *α*-Al_2_O_3_ NPs smaller than 15 nm in the *α*-Al_2_O_3_-NPs-embedded composites during mechanochemical synthesis is thermodynamically unclear. On one hand, the impact of the balls in the high-energy ball milling can bring about a high energy in the collision regions^28^ which may favor the formation of thermodynamically not stable fine equiaxed *α*-Al_2_O_3_ NPs. Metastable metallic, intermetallic and oxide phases can form in high-energy ball milling, as reported in literature^28^. On the other hand, as the Fe_2_O_3_ + 2Al = 2Fe + Al_2_O_3_ reaction appears to take place on atomic scale in the mechanochemical synthesis, the fine equiaxed *α*-Al_2_O_3_ NPs formed in the *α*-Al_2_O_3_-NPs-embedded composites should be surrounded by *α*-Fe, as shown in Fig. 1b. The *α*-Al_2_O_3_/*α*-Fe interfaces in the *α*-Al_2_O_3_-NPs-embedded composites may have an interface energy^29^ lower than the surface energy of the free surfaces of *α*-Al_2_O_3_ NPs, which may stabilise the high interface area of fine equiaxed *α*-Al_2_O_3_ NPs in the *α*-Al_2_O_3_-NPs-embedded composites and conduce to the formation of the fine equiaxed *α*-Al_2_O_3_ NPs smaller than 15 nm. The successful synthesis of disperse fine equiaxed *α*-Al_2_O_3_ NPs smaller 15 nm, which are thermodynamically not stable, exemplifies that thermodynamically not stable nanomaterials may be producible.

The almost fully dense nanocrystalline *α*-Al_2_O_3_ ceramic with a relative density of 99.5% and an average grain size of 60 nm, sintered from the disperse fine equiaxed *α*-Al_2_O_3_ NPs with a narrow size distribution width by two-step pressureless sintering (1,230 °C–1,080 °C for 40 h), exhibits the finest average grain size for a 99.5% dense nanocrystalline *α*-Al_2_O_3_ ceramic (compared with a relative density of 99.5% and an average grain size of 350 nm^10^ or a relative density of 95% and an average grain size of 70 nm^30^) achieved so far by pressureless sintering. Therefore, disperse fine equiaxed *α*-Al_2_O_3_ NPs with narrow size distribution widths, prepared by our mechanochemistry-selective corrosion-refined fractionated coagulation separation approach, show a high sintering activity. Moreover, the successful pressureless sintering of an almost fully dense nanocrystalline *α*-Al_2_O_3_ ceramic with an average grain size as fine as 60 nm, from the disperse fine equiaxed *α*-Al_2_O_3_ NPs with a fine average particle size and a narrow size distribution width, reveals that disperse fine equiaxed *α*-Al_2_O_3_ NPs with a fine average particle size and a narrow size distribution width are an essential prerequisite for the pressureless sintering of dense nanocrystalline *α*-Al_2_O_3_ ceramic with a fine grain size.

In summary, this work presents a simple approach to synthesis of disperse fine equiaxed *α*-Al_2_O_3_ NPs with average particle sizes below 10 nm and narrow size distribution widths. Disperse fine equiaxed *α*-Al_2_O_3_ NPs with average particle sizes below 10 nm and narrow size distribution widths were separated from disperse equiaxed *α*-Al_2_O_3_ NPs with a wide size distribution width by refined fractionated coagulation. Disperse equiaxed *α*-Al_2_O_3_ NPs with a wide size distribution width were prepared by removing the matrixes in the *α*-Al_2_O_3_-NPs-embedded composites through selective corrosion. The *α*-Al_2_O_3_-NPs-embedded composite powders were synthesised by mechanochemical method. The green compacts of *α*-Al_2_O_3_ NPs with an average size of 7.9 nm and a size distribution of 4–14 nm were sintered by a non-optimised two-step pressureless sintering to a almost fully dense nanocrystalline *α*-Al_2_O_3_ ceramic with a relative density of 99.5% and an average grain size of 60 nm, the finest grain size achieved so far by pressureless sintering. Our mechanochemistry-selective corrosion-refined fractionated coagulation separation approach may be scaled up for large-scale production of disperse fine equiaxed *α*-Al_2_O_3_ NPs with average particle sizes below 10 nm and narrow size distribution widths.

## Methods

Fe_2_O_3_ and Al powders mixed stoichiometrically according to Fe_2_O_3_ + 2Al = 2Fe + Al_2_O_3_ were ball-milled in a high-purity argon atmosphere using a high-energy planetary ball mill to synthesise *α*-Al_2_O_3_-NPs-embedded composites. The ball milling conditions were optimised to achieve *α*-Al_2_O_3_ NPs with finest particle sizes and lowest impurity contents as well as for a reasonable production efficiency (See Supplementary Information for experimental details). The optimised ball milling conditions are a main disk rotation speed of 300 rpm, a BPR of 20:1 and a milling duration of 20 h.

To remove the Fe matrixes (and other metal impurities from the vials and balls of the ball mill) in the composite powders and to obtain pure *α*-Al_2_O_3_ NPs, the ball-milled composite powders synthesised by high-energy ball milling were corroded with 12 mol/L hydrochloric acid at room temperature for 10 h and centrifuged. This room temperature acid corrosion was repeated totally three times. Then the powders were corroded with 4 mol/L hydrochloric acid in sealed hydrothermal synthesis reactors at 120 °C for 10 h (See Supplementary Information for experimental details).

In order to obtain disperse fine equiaxed *α*-Al_2_O_3_ NPs with narrow size distribution widths, the disperse equiaxed *α*-Al_2_O_3_ NPs obtained by selective corrosion were size-selectively separated by refined fractionated coagulation using hydrochloric acid as a coagulating agent. The disperse fine equiaxed *α*-Al_2_O_3_ NPs with an average particle size of 14.3 nm and a size distribution width of 2–250 nm were suspended in deionised water in ultrasonic bath and centrifuged at 10000 rpm to remove *α*-Al_2_O_3_ NPs larger than 100 nm. The obtained *α*-Al_2_O_3_ NPs smaller than 100 nm were suspended in a 1.4 mol/L HCl solution, then larger *α*-Al_2_O_3_ NPs coagulated and deposited whereas smaller *α*-Al_2_O_3_ NPs remained stable in the upper clear suspension. The deposited larger *α*-Al_2_O_3_ NPs were centrifuged and used for the next coagulation separation (referred to as the mother powder). A 12 mol/L HCl solution was added into the upper clear suspension; all the *α*-Al_2_O_3_ NPs in the upper clear suspension coagulated and deposited. After centrifuging, washing and drying of the deposited powder, the *α*-Al_2_O_3_ NPs with average particle sizes of 5.2 nm were obtained. In similar ways, the mother powders of the previous refined fractionated coagulation separations were suspended in order in the HCl solutions of 1.2, 1.0 and 0.8 mol/L concentrations, and the *α*-Al_2_O_3_ NPs with average particle sizes of 6.5, 7.9 and 9.6 nm were separated respectively.

The *α*-Al_2_O_3_ NPs with an average particle size of 7.9 nm and a size distribution width of 4–14 nm were pressed into green compacts at 600 MPa. The green compacts were heated in air at a heating rate of 10 °C/min to 1,230 °C without hold, then cooled at a rate of 5 °C/min down to 1,080 °C with a 40 h hold and finally cooled at a rate of 10 °C/min down to room temperature.

Phases in *α*-Al_2_O_3_-NPs-embedded composite powders, *α*-Al_2_O_3_ NPs obtained by removing the Fe matrix in the composites and *α*-Al_2_O_3_ NPs size-selectively separated by refined fractionated coagulation were examined by XRD analysis. The morphology, microstructure and lattice structure of *α*-Al_2_O_3_-NPs-embedded composite powders, *α*-Al_2_O_3_ NPs obtained by removing the Fe matrix in the composites and *α*-Al_2_O_3_ NPs size-selectively separated by refined fractionated coagulation were analysed by TEM (or HRTEM) observations. SAED analysis was performed during TEM analysis. The average particle size and size distribution of the *α*-Al_2_O_3_ NPs obtained by removing the Fe matrix in the composites were statistically determined from more than 5000 particles observed in the TEM images of the different areas of the samples. The average particle sizes and size distributions of the *α*-Al_2_O_3_ NPs size-selectively separated by refined fractionated coagulation were statistically determined from more than 1500 particles observed in the TEM images of the different areas of the samples. The compositions of *α*-Al_2_O_3_ NPs were determined by EDS and ICP-AES elemental analyses. The specific surface areas of the *α*-Al_2_O_3_ NPs were measured by N_2_ adsorption at 77 K using the BET method. Before the BET measurements, the powder samples were degassed at 200 °C for 5 h. Relative densities of the green compacts and sintered bodies were measured by Archimedes' method. Microstructure of sintered bodies was analysed by SEM observations. The average grain size and grain size distribution of sintered nanocrystalline *α*-Al_2_O_3_ ceramic samples were statistically determined from about 1000 grains observed in the SEM images of the different areas of the samples.

## Additional Information

**How to cite this article**: Pu, S. *et al*. Disperse fine equiaxed alpha alumina nanoparticles with narrow size distribution synthesised by selective corrosion and coagulation separation. *Sci. Rep*. **5**, 11575; doi: 10.1038/srep11575 (2015).

## Supplementary Material

Supplementary Information

## Figures and Tables

**Table 1:** Average particle sizes and size distribution widths of the *α*-Al_2_O_3_ NPs separated by refined fractionated coagulation separation at different HCl concentrations.

HCl concentration (mol/L)	Average particle size (nm)	Size distribution width (nm)
1.4	5.2	2–9
1.2	6.5	3–11
1.0	7.9	4–14
0.8	9.6	5–15
