# Supplementary material for: Disperse fine equiaxed alpha alumina nanoparticles with narrow size distribution synthesised by selective corrosion and coagulation separation
Source: Sci Rep. 2015 Jul 13;5:11575. doi: 10.1038/srep11575 (PMC4648409; doi:10.1038/srep11575)
Supplement: Supplementary Information [file srep11575-s1.pdf]

Supplementary Information

Disperse fine equiaxed alpha alumina nanoparticles with narrow size distribution synthesised by selective corrosion and coagulation separation

Sanxu Pu, Lu Li, Ji Ma, Fuliang Lu† & Jiangong Li*

Institute of Materials Science & Engineering, Lanzhou University, Lanzhou 730000, China

† Present address: Zhejiang Crystal-Optech Co. Ltd., Yingtan, Jiangxi 335000, China

Correspondence: Professor Jiangong Li, Institute of Materials Science & Engineering, Lanzhou University, Lanzhou 730000, China. E-mail: lijg@lzu.edu.cn

1. Materials and Instrumentation

All the chemicals were used as received, including Al powders (≥99.0 wt.%, 100-200 meshes, Sinopharm), Fe_2O_3 powders (≥99.0 wt.%, 0.2-0.3 μm, Sinopharm), hydrochloric acid (36.0-38.0 wt.% in water, Tianjin Fuyu) and ethanol absolute (≥99.7 wt.%, Guangdong Xilong).

High-energy ball milling was performed in two round-bottom stainless steel vials (80 mL each) with stainless steel balls of 10 mm in diametre on a Fritsch P4 planetary ball mill. Centrifugation was performed on a centrifuge (TG22) with rotation speeds tunable from 100 to 22000 rpm. X-ray diffraction (XRD) patterns (2θ scans) were recorded using an x-ray diffractometer (Rigaku D/Max-2400) with Cu K_α radiation (λ=0.154050 nm) and a scanning speed of 5°/min working with a voltage of 40 kV and a current of 150 mA. Scanning electron microscopy (SEM) observations were conducted by a field emission scanning electron microscope (Hitachi S4800) working at a voltage of 3 kV. Transmission electron microscopy (TEM) observations as well as selected area electron diffraction (SAED) and energy dispersive x-ray spectroscopy (EDS) analyses were performed on a field emission transmission electron microscope (FEI Tecnai G^2 F30) working at a voltage of 300 kV. Inductively coupled plasma-atomic emission spectrometry (ICP-AES) elemental analysis was conducted on a TJA IRIS ER/S ICP-AES spectrometer. Specific surface areas of α-Al_2O_3 nanoparticles were measured on a Micrometrics ASAP 2020M surface area analyser.

2. Synthesis of composites of α-Al_2O_3 nanoparticles (NPs) embedded in the Fe matrix

Powders of Fe_2O_3 and Al were mixed stoichiometrically according to

$$Fe_2O_3 + 2Al = 2Fe + Al_2O_3 \quad (1)$$

and loaded in the vials along with the balls in a high-purity argon atmosphere.

The powder mixtures were milled for different milling durations at a main disk rotation speed of 300 rpm, a relative rotation speed ratio of the vial to the main disk of -2 and a ball-to-powder ratio (BPR) of 20:1. The total mass of the starting powders is about 5 g. For a milling duration of 8 h, the milled powders consist of α-Al_2O_3 and α-Fe with a small amount of γ-Al_2O_3. The powders milled for 20 h contains only α-Al_2O_3 and α-Fe, as revealed by XRD analysis (**Fig. 1a**). After ball milling, the milled powders were corroded with hydrochloric acid (details to be described in Supplementary Information Part 3) to obtain α-Al_2O_3 NPs. The average particle size of the obtained α-Al_2O_3 NPs (determined by TEM observations) decreases a little at first and then increases slightly with increasing milling duration from 20 to 100 h.

As the BPR increases from 20:1 to 50:1 for a main disk rotation speed of 300 rpm and a milling duration of 20 h, the average particle size of the obtained α-Al_2O_3 NPs decreases from 14.3 to 10.7 nm. However, the Fe impurity content in the α-Al_2O_3 NPs increases up to 0.9% (mass percent). Besides, a high BPR reduces the loading amount of the starting powders and thus lowers the efficiency of ball milling for preparing α-Al_2O_3 NPs.

As the main disk rotation speed increases from 200 to 300 rpm for a BPR of 20:1 and a milling duration of 20 h, the γ-Al_2O_3 phase in the obtained α-Al_2O_3 NPs decreases and disappears. Meanwhile, the average particle size of the α-Al_2O_3 NPs decreases.

According to the series of the above ball-milling experiments, the ball milling conditions were optimised for preparing α-Al_2O_3 NPs with finest particle sizes and lowest impurity contents as well as for a reasonable production efficiency.

3. Preparation of disperse equiqaxed α-Al_2O_3 NPs

The composite powders (α-Al_2O_3 NPs embedded in the Fe matrix) synthesised by ball milling under the optimised milling conditions were put into 12 mol/L hydrochloric acid at room temperature, stirred for 10 h and centrifuged at 10000 rpm. After three times room temperature HCl corrosion, the powders were transferred into 4 mol/L HCl in 50 ml hydrothermal synthesis reactors of Teflon lined stainless steel. The hydrothermal synthesis reactors with the powders in 4 mol/L HCl were sealed, heated at 120°C for 10 h in an oven and then cooled down to room temperature in air. The precipitates in the hydrothermal synthesis reactors were centrifuged at 10000 rpm, washed several times with 4 mol/L HCl in an ultrasonic bath, centrifuged and dried at 80°C for 5 h in an oven. The SAED pattern of the obtained powders shown in Supplementary **Fig. S1a** indicates the pure α-Al_2O_3 phase. A low magnification TEM image of the powders is shown in **Fig. S1b**. The EDS analysis reveals that the α-Al_2O_3 NPs are Al_2O_3 with Fe impurity (Supplementary **Fig. S2** and **Table S1**). ICP-AES elemental analysis shows 99.3334% (mass percent) Al, 0.5106% Fe and 0.1559% Cr in the α-Al_2O_3 NPs (Supplementary **Table S2**). Combining the EDS and ICP-AES analyses, the purity of the α-Al_2O_3 NPs was determined to be 99.646% (mass percent) with 0.271% Fe and 0.083% Cr.

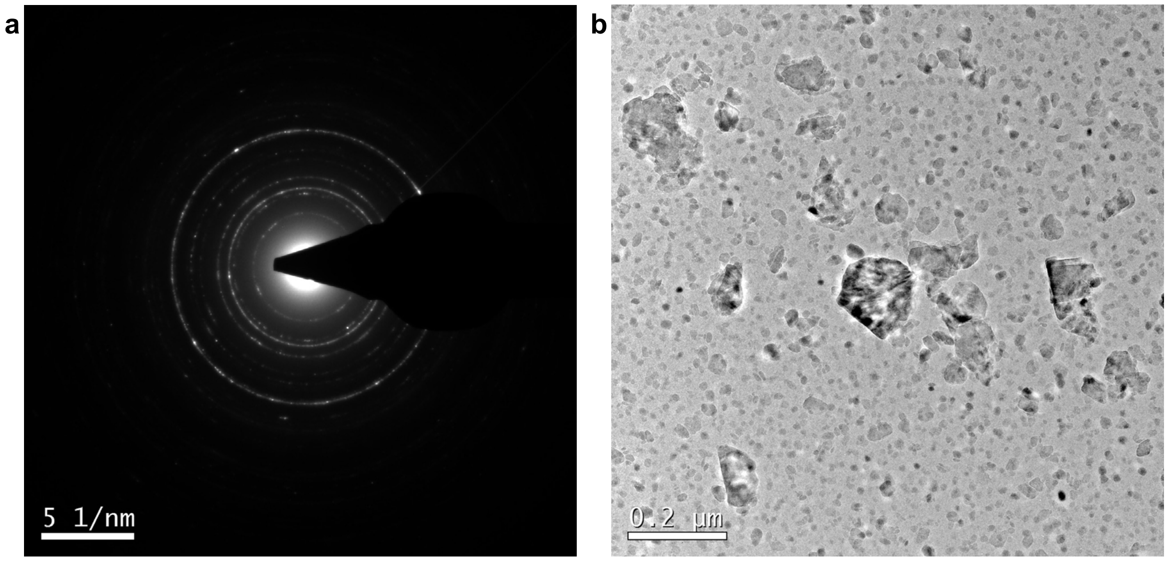

Figure S1: (a) SAED pattern of the α-Al_2O_3 NPs obtained by removing the Fe matrix in the composites of α-Al_2O_3 NPs embedded in the Fe matrix through HCl corrosion, showing a typical SAED pattern of α-Al_2O_3. (b) Low magnification TEM image of the α-Al_2O_3 NPs obtained by removing the Fe matrix in the composites, showing disperse equiaxed α-Al_2O_3 NPs of sizes ranging from 3 to 200 nm.

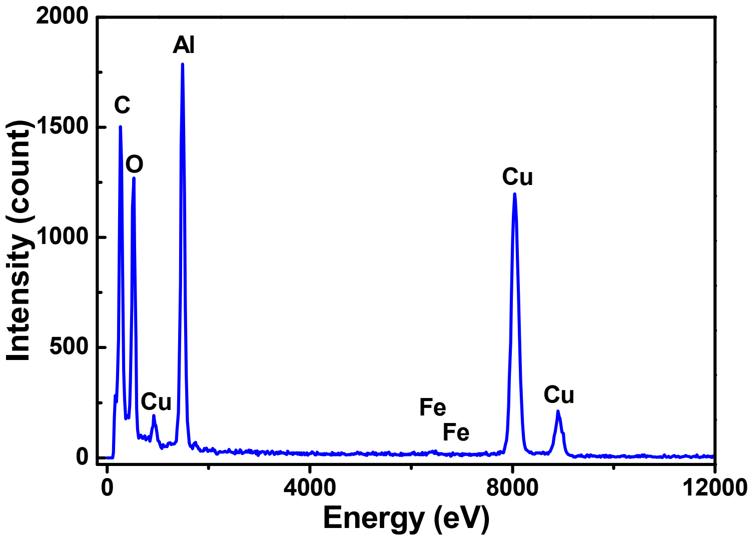

Figure S2: EDS spectrum of the α-Al_2O_3 NPs obtained by removing the Fe matrix in the composites of α-Al_2O_3 NPs embedded in the Fe matrix through selective corrosion. Copper and carbon are from the copper grids and the carbon films on the copper grids holding the α-Al_2O_3 NPs for the TEM analysis.

Table S1: EDS analysis results for the α-Al_2O_3 NPs obtained by removing the Fe matrix in the composites of α-Al_2O_3 NPs embedded in the Fe matrix through selective corrosion

Element	Mass %	Atomic %
O (K)	53.20	65.84
Al (K)	46.30	33.97
Fe (K)	0.49	0.17

Table S2: ICP-AES elemental analysis results for the α-Al_2O_3 NPs obtained by removing the Fe matrix in the composites of α-Al_2O_3 NPs embedded in the Fe matrix through selective corrosion

Element	Average (mg/L)	Mass %
Al	551.1000	99.3334
Fe	2.8330	0.5106
Cr	0.8651	0.1559

4. Preparation of disperse fine equiaxed α-Al_2O_3 NPs with narrow size distribution widths by refined fractionated coagulation separation

Disperse equiaxed α-Al_2O_3 NPs with an average particle size of 14.3 nm and a size distribution width of 2-250 nm were size-selectively separated using HCl as a coagulating agent, by decreasing the HCl concentration in a small interval.

Supplementary **Fig. S3a** shows a low magnification TEM image of the α-Al_2O_3 NPs with an average particle size of 5.2 nm and a size distribution width of 2-9 nm separated with 1.4 mol/L HCl.

Supplementary **Fig. S3b** shows a low magnification TEM image of the α-Al_2O_3 NPs with an average particle size of 6.5 nm and a size distribution width of 3-11 nm separated with 1.2 mol/L HCl.

Supplementary **Fig. S3c** shows a low magnification TEM image of the α-Al_2O_3 NPs with an average particle size of 7.9 nm and a size distribution width of 4-14 nm separated with 1.0 mol/L HCl.

Supplementary **Fig. S3d** shows a low magnification TEM image of the α-Al_2O_3 NPs with an average particle size of 9.6 nm and a size distribution width of 5-15 nm separated with 0.8 mol/L HCl.

Supplementary **Fig. S3e** and **S3f** show high and low magnification TEM images of the α-Al_2O_3 NPs with an average particle size of 11.3 nm and a size distribution width of 6-19 nm separated with 0.6 mol/L HCl.

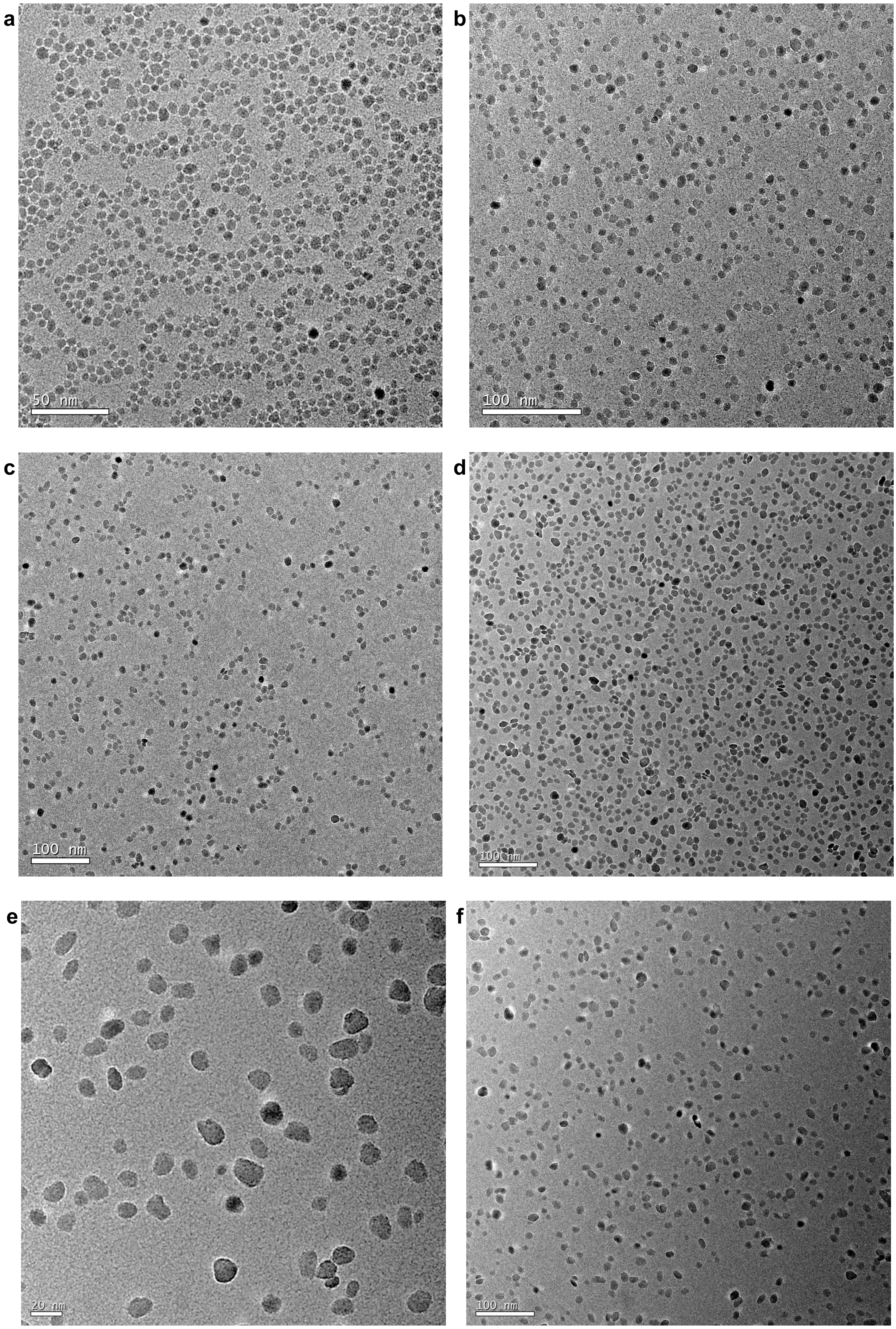

Figure S3 (to be continued)

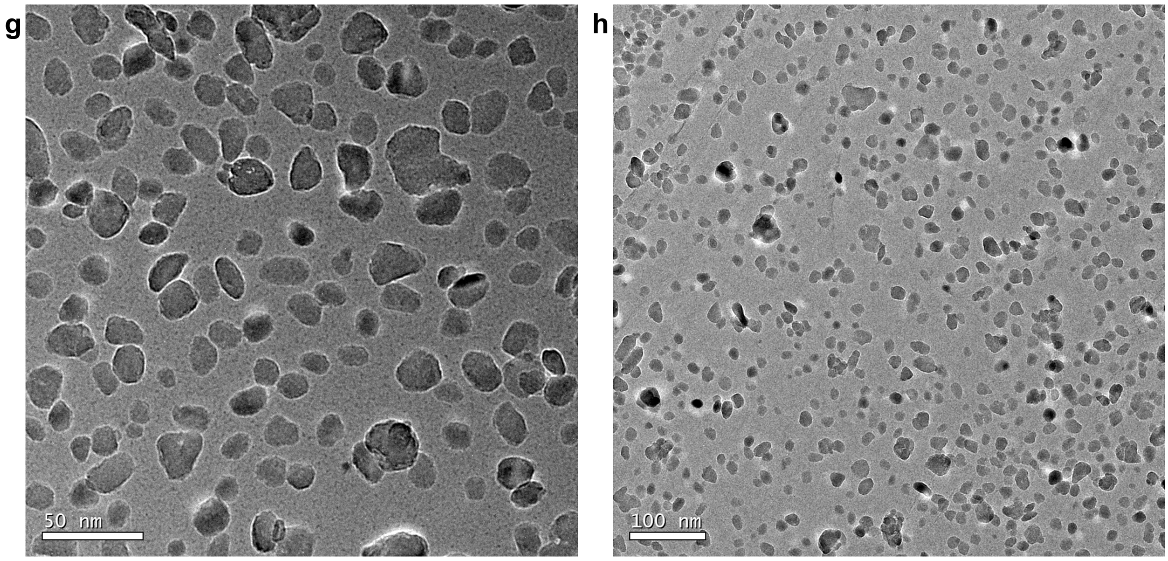

Figure S3: TEM images of the α-Al_2O_3 NPs with average sizes of 5.2 (**a**), 6.5 (**b**), 7.9 (**c**), 9.6 (**d**), 11.3 (**e,f**) and 14.8 nm (**g,h**) separated by refined fractionated coagulation separation with 1.4, 1.2, 1.0, 0.8, 0.6 and 0.4 mol/L HCl respectively.

Supplementary **Fig. S3g** and **S3h** show high and low magnification TEM images of the α-Al_2O_3 NPs with an average particle size of 14.8 nm and a size distribution width of 8-32 nm separated with 0.4 mol/L HCl.

Supplementary **Fig. S4** shows the Size distribution histogram of the α-Al_2O_3 NPs with an average size of 7.9 nm (TEM images in **Fig. 3e** and Supplementary **Fig. S3c**) separated with 1.0 mol/L HCl.

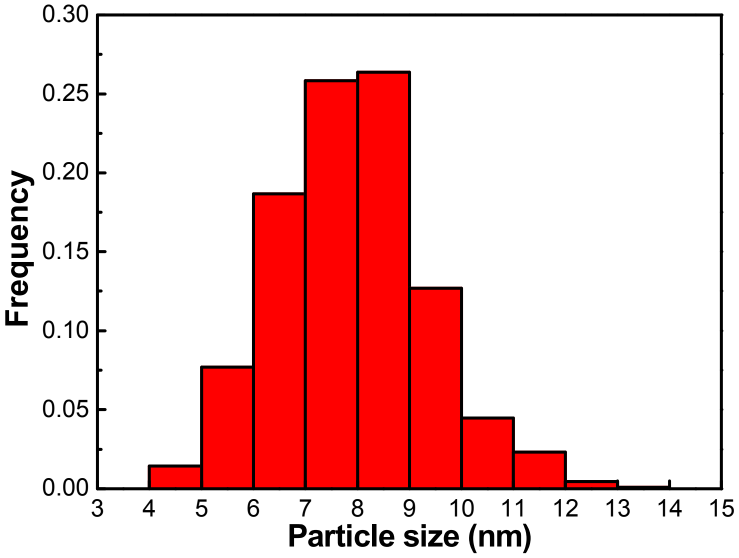

Figure S4: Size distribution histogram of the α-Al_2O_3 NPs with an average size of 7.9 nm (TEM images in Fig. 3e and Supplementary Fig. S3c) obtained by refined fractionated coagulation separation with 1.0 mol/L HCl.

As an example, Supplementary **Fig. S5** shows the XRD pattern of the α-Al_2O_3 NPs with an average size of 7.9 nm obtained by refined fractionated coagulation separation, showing the broad diffraction peaks of the α-Al_2O_3 NPs.

The BET plot for the α-Al_2O_3 NPs with an average size of 7.9 nm (obtained by refined fractionated coagulation separation) measured by N_2 adsorption at 77 K is plotted in Supplementary **Fig. S6**.

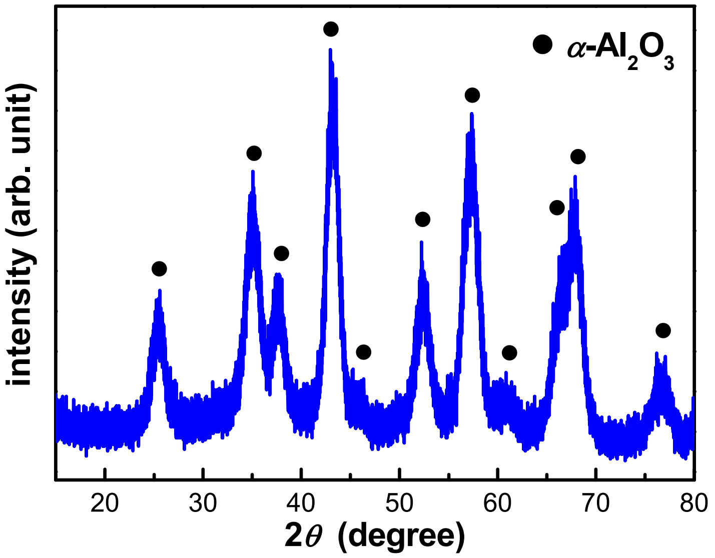

Figure S5: XRD pattern of the α-Al_2O_3 NPs with an average size of 7.9 nm obtained by refined fractionated coagulation separation with 1.0 mol/L HCl, showing the broad diffraction peaks of the α-Al_2O_3 NPs.

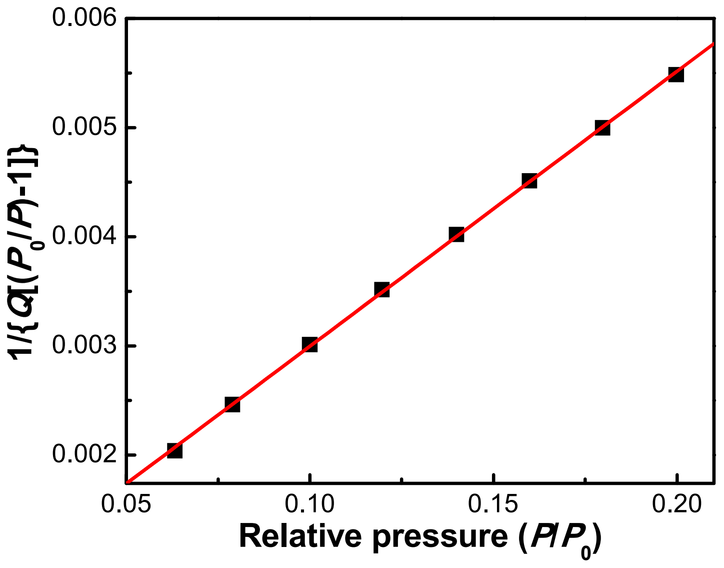

Figure S6: BET plot of the α-Al_2O_3 NPs with an average size of 7.9 nm using points collected in a range of relative pressures P/P_0 from 0.06 to 0.2. Q is the volume of gas adsorbed by the powder sample at a relative pressure P/P_0 (in cm^3/g).

5. Sintering of nanocrystalline α-Al_2O_3 ceramic from disperse fine equiaxed α-Al_2O_3 NPs with narrow size distribution

The green compacts pressed from α-Al_2O_3 NPs with an average particle size of 7.9 nm and a size distribution width of 4-14 nm at 600 MPa were sintered in air by a non-optimised two-step sintering (1,230°C without hold and 1,080°C with a 40 h hold). Sintered bodies were broken; the cross sections of the sintered bodies were polished and thermally etched at 1,050°C. The microstructure of the sintered bodies was analysed by SEM. Supplementary **Fig. S7** shows a low magnification SEM image of the sintered bodies.

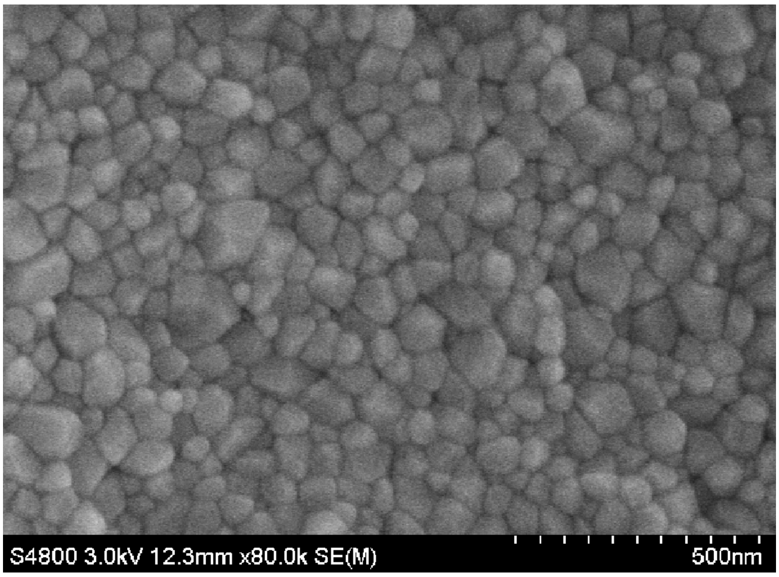

Figure S7: Low magnification SEM image of the sintered body for a green compact pressed from disperse fine equiaxed α-Al_2O_3 NPs with an average size of 7.9 nm and a size distribution width of 4-14 nm at 600MPa and sintered in air by a two-step sintering (heating to 1,230°C without hold and decreasing to 1,080°C with a 40 h hold) (after additional thermal etching).
